# The True Self. Critique, Nature, and Method

**DOI:** 10.3389/fpsyg.2019.02250

**Published:** 2019-10-22

**Authors:** Terje Sparby, Friedrich Edelhäuser, Ulrich W. Weger

**Affiliations:** ^1^Department of Psychology and Psychotherapy, University of Witten/Herdecke, Witten, Germany; ^2^Department of Psychology, Integrated Curriculum for Anthroposophic Psychology (ICURAP), University of Witten/Herdecke, Witten, Germany; ^3^Department of Medicine, Integrated Curriculum for Anthroposophic Medicine (ICURAM), Witten/Herdecke University, Witten, Germany; ^4^Department of Medicine, Institute of Integrative Medicine, Witten/Herdecke University, Witten, Germany

**Keywords:** the true self, the self, first-person methods, consciousness, phenomenology

## Abstract

The history of philosophy gives us many different accounts of a true self, connecting it to the essence of what a person is, the notion of conscience, and the ideal human being. Some proponents of the true self can also be found within psychology, but its existence is mostly rejected. Many psychological studies, however, have shown that people commonly believe in the existence of a true self. Although folk psychology often includes a belief in a true self, its existence is disputed by psychological science. Here, we consider the critique raised by Strohminger et al., stating that the true self is (1) radically subjective and (2) not observable, hence cannot be studied scientifically (Strohminger et al., 2017). Upon closer investigation, the argument that the self is radically subjective is not convincing. Furthermore, rather than accepting that the true self cannot be studied scientifically, we ask: What would a science have to look like to be able to study the true self? In order to answer this question, we outline the conceptual nature of the true self, which involves phenomenological and narrative aspects in addition to psychological dimensions. These aspects together suggest a method through which this concept can be investigated from the first-person perspective. On a whole, we propose an integrative approach to understanding and investigating the true self.

## Introduction

Let us start with a quote: “Many people like to think they have an inner “true” self. Most social scientists are skeptical of such notions. If the inner self is different from the way the person acts all the time, why is the inner one the “true” self?” (Baumeister and Bushman, 2013, p. 75). This is how the notion of a true self is introduced in a recent textbook on social science. It is suggested that there is a conflict between folk psychology and science, where the true self is a notion that does not hold up to closer scrutiny. This view has recently been reinforced by a number of studies conducted by Strohminger and Nichols (2014) and Strohminger et al. (2017), showing that belief in a true self is indeed common while questioning its actual existence. Is the view we have of our “true self” merely a reflection of the socio-cultural environment in which we exist? And can someone have a “true self” that is good, even if they continually act in ways that are harmful?

Positing a chimera of an inherently good “true self,” existing so deeply within the structure of someone’s psyche that it may never make an appearance in reality may seem completely unwarranted. Not only does this put the true self beyond scientific observation, it also makes it seem like a hopelessly optimistic dream. Hence, although it is empirically clear that people make use of the concept of a true self – in the sense of that which cannot change without someone becoming less of what they really are – there are weighty reasons to doubt whether the true self exists beyond the widespread belief in it. Since this belief is so common, could it be that it is in fact grounded in reality?

This is the question that we will explore in the following, outlining not only a suggestion for what the structure of the true self might be but also sketching out a method for investigating it. In doing this, we will also provide counter-arguments to the critique of the aforementioned true self. In our view, the true self can be viewed as having a kind of spiritual existence. It can appear in time but also exists beyond time. It may even be absent at different moments in time without ceasing to exist. Complete absence of the true self would, however, make it impossible to investigate. We take it that we are dealing with an essence of the Hegelian kind, i.e. an essence, the essence of which is to appear (and indeed, can there be an essence that never appears?). In other words, the true self cannot be so chimeric as to never enter the stage of actual life. However, such an object of study cannot be investigated adequately using conventional philosophical or psychological methods alone. We propose that the true self may be approached through a first-person method combining both philosophical reflection and introspective observation, as we will outline in section “Outline of a Comprehensive First-Person Method for Studying the True Self.” Before introducing this method, we will look into the history and nature of the self and the true self in philosophy and psychology (section “Introduction”). This will follow with a response to critiques of the true self (section “The Problem of Radical Subjectivity and Observability of the True Self”).

### A Short Historical Account of the Self and the True Self

The self, one of the most central as well as critically discussed concepts in philosophy and psychology, has a long history. The idea that one has an underlying self in addition to a surface personality can be traced back to the notion that one has a soul that is potentially immortal. In the Egyptian culture, only the Pharaoh possessed an immortal, divine soul (akh) while alive. Only at the moment of death could other Egyptians gain such a soul (Waage, 2008). In Ancient Greek culture, Socrates was known for having heard an inner voice that indicated to him what he should (*Memorabilia* 1.1.4, 4.3.12, 4.8.1, *Apology* 12) and should not do (*Apology* 31c-d, 40a-b, *Euthydemus* 272e-273a). This was part of what lead to his demise, as he was accused of following other gods. The inner voice was a *daimonion*, a divine being (particular) to Socrates and not one of the gods condoned by the Athenian city-state. Such a private divine being is now commonly understood to refer to conscience in the Christian tradition (Schinkel, 2007, p. 97), which is connected to the moral essence – the true self – of an individual. The idea of a person’s moral essence was developed further in Greek thought. For example, it was connected to the performance of specific virtues by Aristotle. Aristotle also suggested that “the true self of each” is the divine intellect or *nous* (NE, 1178, a2).

However, when answering the question “who are you?”, it was for a long time customary to name one’s ancestors. In ancient Rome, the firstborn son was the property of the *pater familias* until the death of the father. During the funeral procession, the son wore the father’s death mask (Salemonsen, 2005). It may be noted that the word “mask” (*lat.* persona) is related to the word “person,” suggesting that we can take on different identities but also that there is an underlying essence. Augustus is known for writing the first autobiography, inaugurating a genre defined by the idea that certain events and thoughts are more important than others when seeking to understand who someone is. Arguably, the Judeo-Christian religions also contributed to the view that all human beings have a divine core, regardless of background: “There is neither Jew nor Greek, there is neither bond nor free, there is neither male nor female: for ye are all one in Christ Jesus” (Galatians 3:28). In the Renaissance, Pico della Mirandola emphasized the notion of agency in his “Oration on human dignity”, making God exclaim that it is a matter of will whether the human being shall become animal or divine, mortal or immortal:

I have placed you at the very center of the world, so that from that vantage point you may with greater ease glance round about you on all that the world contains. We have made you a creature neither of heaven nor of earth, neither mortal nor immortal, in order that you may, as the free and proud shaper of your own being, fashion yourself in the form you may prefer. It will be in your power to descend to the lower, brutish forms of life; you will be able, through your own decision, to rise again to the superior orders whose life is divine (della Mirandola, 1996, p. 7).

For Kant, the self is that which provides transcendental unity to our thoughts and perceptions, in short, to all our experiences (Kant, 1904). Although the self cannot be known as it is in itself, in Kantian ethics, the individual is fully autonomous, free, when it acts according to rational principles (Kant, 1968). The individual manifests the kingdom of heaven on earth to the extent that ethical principles are adhered to as if they were natural laws. As a reaction to this, some philosophers, such as Sartre, point out that this view disregards the communal and social aspects of the self as well as its individuality and authenticity (Sartre, 2014). Rejecting Sartre’s notion of authenticity, Foucault denied that there is any self that is given to us; claiming that we should rather view the self as a work of art:

I think that from the theoretical point of view, Sartre avoids the idea of the self as something that is given to us, but through the moral notion of authenticity, he turns back to the idea that we have to be ourselves – to be truly our true self. I think that the only acceptable practical consequence of what Sartre has said is to link his theoretical insight to the practice of creativity – and not that of authenticity. From the idea that the self is not given to us, I think that there is only one practical consequence: we have to create ourselves as a work of art (Focault, 1997, p. 262).

Foucault points out that Sartre’s notion of authenticity reintroduces a given measure for someone’s true self. Foucault thinks we should be more radical in our rejection of any given content or measure of what constitutes the true self. Any such content or measure we must create ourselves. One may remark that even creative acts contain an element of or at least relate to something given, for example an inspiration or a framework of understanding. The idea of creating a self does not need to be thought of as a pure/arbitrary invention of something incomprehensible. Creative acts may be understood instead as the encounter between something given and subjective energy. In part, the subject identifies with the given, subjects itself to it, and in part, the subject recognizes the given as itself.

If we pause and summarize here, we can see that there is a whole host of ideas connected to the self in the Western canon (for a discussion of self, no-self and true self accounts in Asian traditions, see Siderits et al., 2010):

1. The self is a kind of essence, substance, or a soul that may or may not survive death
2. The self is the voice of conscience, the source of moral or authentic action
3. The self is divine, possibly created by God
4. The self is related to the past, to ancestry, and outward identity such as one’s work
5. The self has a story connected to it that can be represented in a biography
6. The self provides unity to cognition and experience
7. The self is a free, autonomous agent
8. The self is essentially connected to other human beings and culture
9. The self has to be created

As we can see from this short and non-exhaustive list, the self is complex and may be conceived in conflicting ways. For example: Is the self-created by God or the individual? Is the self completely autonomous or is it thoroughly culturally determined? Is the self an essence or is it a story? None of these are necessarily contradictory, but much work is required to flesh out a comprehensive conception of the self. Do all these characteristics have something in common? This question is not easy to answer. If we cannot find a common characteristic in all the different definitions, we may have to concede that the self is simply a name for a host of unrelated ideas or aspects of human existence. On a closer look, each item on the list can potentially be said to be the true self. Even one’s outward identity could arguably be seen to constitute a true self. Imagine a *puer aeternus*, a Peter Pan-like existence: someone who is reluctant about identifying with anything at all, preferring to stay adolescent indefinitely. For such a person, actually identifying with something could be said to be a realization of their true self (their true self would not necessarily be the specific outward identity but could be manifested by taking on a concrete and not fantastical identity). There is one way of conceiving what the nature of the true self is, which we will elaborate in the following, that does not imply that we have to make a choice about which specific self represents the true one. This is the conception of the true self as a whole that unifies the different selves. Moreover, the true self can not only be viewed as a whole but also as the manifestation of a specific moral self that has grown out of the past. The true self, on this conception, has both distanced itself from the past and integrated it, moving toward an ideal that is in one sense given, internally and from the past, but in another sense must also be created, or is only just coming into existence from the future.

### The True Self in Philosophy and Psychology

Although the existence of the self is controversial in philosophy (Metzinger, 2003; Siderits et al., 2010; Ganeri, 2012), there are a number of influential philosophers who claim that there at least a minimal or core self exists. Such a view can be found both among traditional thinkers, such as Descartes, Leibniz, Kant, Hegel, Husserl, etc., and contemporary ones (MacIntyre, 1981; Taylor, 2012; Zahavi, 2017). Charles Taylor has specifically addressed the notion of a true self in the context of a discussion of negative and positive freedom (Taylor, 1985; Sparby, 2014). Negative freedom is the idea that one can realize one’s true self insofar as there are no external restrictions on the self (and perhaps no internal restrictions such as fear). But where does the understanding of what actually counts as being the true self come from? If it comes, for instance, from a totalitarian state, then the “true self” may indeed be a false self since someone other than the self determines it. Hence it would follow that actualizing a true self is typically seen to include self-determination. It could of course be that content of a state prescribed true self accords *by coincidence* with the true self recognized by a person. This would not stop the person from actualizing the true self as long as the recognition is internally constituted through reflection and moral deliberation. However, if someone can determine themselves radically, does this not mean that the content of the true self is arbitrary? We believe that such problems can be solved with ideas as such “being-with-oneself in otherness” (Sparby, 2016). For example, acting according to one’s true self does not exclude acting according to principles as long as these principles are recognized as stemming from the true self. Finding one’s true self may involve finding oneself in another person, community, culture, etc. This does not mean that the true self is simply something given. Even creative processes can involve something approaching the self “from the outside”, such as an inspiration. Again, the true self can be viewed as a whole, as something transcending the subject-object dichotomy, allowing for such events where something comes to the self seemingly from an external source (e.g. the voice of conscience), a source which is, however, more adequately conceived of as belonging to the self in a deeper, higher or more inclusive sense. It is of course possible that the voice of conscience might be an expression of an internalized dogmatic morality. However, this does not make it unreliable in principle. It means that what it dictates has to be viewed in light of an investigation of what its source might be, considering cultural factors specifically.

Is a person always acting in accordance with their true self if they act according to their self? The problem here is that the self is not only multifaceted but also contradictory given that different aspects are in conflict with each other. For example, the human being can act out of principle or according to their desires. Both may be viewed or at least experienced as essential parts of one’s identity, although these parts do not always harmonize. If one acts according to one’s desire, another desire may not be fulfilled. If one acts morally, desires may fail to be satisfied at all. If one acts in a case where there is a moral dilemma, the true self seems to be constituted by that act. But what if I act based on wrong information, inherited cultural views, or delusion? Indeed, as we shall see, one of the main critiques of the true self is its radical subjectivity. The beliefs and actions that we ascribe to the true self depend on our worldview that is ultimately a reflection of the culture we belong to.

The field of psychology has contributed to our understanding of the self by gathering empirical support for the view that we are indeed ruled by external forces, such as unconscious desires, bias, and social conditioning. It has been shown that the experience of living a meaningful life is associated with having cognitive access to one’s true self, and yet psychological research remains either skeptical or agnostic about the existence of it (Schlegel et al., 2013) despite the belief in a true self seems to be independent of personality type and culture (De Freitas et al., 2018). However, one can indeed find representatives of notions of a true self also in psychology. The true self is sometimes referred to as the I-self or self-as-process as opposed to the me-self or self-as-object (Ryan and Rigby, 2015). The former “concerns the conceptions, images, roles, statuses, and attributes associated with an identity,” while the latter “concerns the inherent integrative tendencies of people to understand, grow, and create coherence in their experiences” (Ryan and Rigby, 2015, p. 246). The psychoanalyst Winnicott made explicit use of the concept of a true self, contrasting it with the false self (Winnicott, 1965). His view of the true self can be summarized as the self that is spontaneous, alive, and creative – the false self would then be a persona that lacks those characteristics (Rubin, 1998, p. 102). Numerous other terms are used for the true self such as the real self, the ideal self, the authentic self, the intrinsic self, the essential self, and the deep self [see overview of sources in Strohminger et al. (2017)]. Strohminger et al. have shown that people on average understand moral traits to be most fundamental to a person in addition to personality, memories, and desires, while characteristics related to perceptual abilities (e.g. near-sightedness) and psychical traits are perceived as having the least impact on who someone essentially is (Strohminger and Nichols, 2014). The essential differences between the self and the true self according to Strohminger et al. are that the self (1) encompasses the entire range of personal features, (2) is valence independent (it is inherently neither good nor bad) but (3) is perspective (first- or third-person) dependent, and (4) is cross-culturally variable, while the true self has an emphasis on (1) moral features, is (2) valence-dependent or positive by default, (3) perspective independent, and (4) cross-culturally stable (Strohminger et al., 2017, p. 3).

Strohminger et al. have also provided a particularly powerful formulation of the argument against the true self, which is quoted in full since it is the critique used as the background to our suggestion of what the nature of the true self is and how it can be studied:

Is the true self also a scientific concept, one that can be used to describe how the mind actually works? Is there, in other words, a true self? The evidence reviewed here points to two properties relevant to this question. One: the true self depends on the values of the observer. If someone thinks homosexual urges are wrong, she will say the desire to resist such urges represents the true self (Newman et al., 2014). And if she scores high in psychopathy, she will assign less weight to moral features in her conceptualization of personal identity (Strohminger and Nichols, 2014). What counts as part of the true self is subjective, and strongly tied to what each individual person herself most prizes.

Two: The true self is, shall we say, evidence-insensitive. Resplendent as the true self is, it is also a bashful thing. Yet people have little trouble imbuing it with a host of hidden properties. Indeed, claims made on its behalf may completely contradict all available data, as when the hopelessly miserable and knavish are nonetheless deemed good “deep down”. The true self is posited rather than observed. It is a hopeful phantasm.

These two features—radical subjectivity and unverifiability—prevent the true self from being a scientific concept. The notion that there are especially authentic parts of the self, and that these parts can remain cloaked from view indefinitely, borders on the superstitious. This is not to say that lay belief in a true self is dysfunctional. Perhaps it is a useful fiction, akin to certain phenomena in religious cognition and decision-making (Gigerenzer and Todd, 1999; Boyer, 2001). But, in our view, it is a fiction nonetheless (Strohminger et al., 2017, p. 7).

To reiterate, the problem facing the true self-view is that it is a conception tied to the values of a person, which are determined subjectively according to the structure of their personality, and by the culture and social environment in which that person exists. What the authors mean by “radical subjectivity” is, however, not clear. Does it mean that the values that a person uses to measure whether they live up to their true self are arbitrary, that the true self is based on a radical existential choice not grounded in anything, or that it is determined by biological, cultural, or social factors that happen to affect the person? These are issues that need to be untangled and answered. Furthermore, a good response is needed when arguing that the true self is not observable and therefore fictional. In particular, does it make sense to speak of a true self if that self never manifests? Can a person be called inherently good if they commit heinous crimes and consistently act in ways that are harmful to others, taking pleasure in their suffering?

In order to argue in favor of the existence of the true self, one must address the critique that it is a radically subjective notion and that it is unverifiable. Since we take the view that the self is not a thing with clearly defined borders but rather an organizing principle of a continual process, speaking of “the existence” of a true self can be misleading. Nevertheless, one may claim that there is such an organizing principle and that the true self is neither radically subjective nor unverifiable. Before turning to that, we will provide a preliminary delineation of the true self that we will flesh out as we address the critique above.

### A Thin and Thick Conception of the True Self and Their Unity

Two conceptions of the true self are implicit in what has been said above, which we will refer to as the thin and thick conception of the true self. One way to characterize them is to say that the thin conception is static: unchangeable, timeless, always the same. The thick conception is dynamic: developing, spread out over long changes of time, and continually emerging. The current objective in the following is to unite these two conceptions (in fact, to show how they are interdependent) and to investigate how such an account may be able to respond to the critique raised against the true self that we will focus on in section “The Problem of Radical Subjectivity and Observability of the True Self”.

The thin conception of the true self is the idea that the self has a deeper and more essential nature; the true self is identical to this essential part of the self. Some of the properties attached to the self are accidental while others are essential. Someone can change their job and although they may have identified with their job, they do not really cease to be who they truly are when they change jobs. The true self as the essential self can consist of either one essential property or a set of properties. Sometimes, this is also referred to as the minimal self, which can be defined as the simple quality of subjective experience; the most fundamental experience of what it is like to be this or that subject (Zahavi, 2017). However, as pointed out by Fasching, the essential self’s nature may be exactly a *bare existence*; not recognizable by any property. It simply is and we know it as something that can identify itself with potentially anything but can never be reduced to any specific property (Fasching, 2016). A similar view is presented by Ramm, who, using first-person experiments, argues that the self in itself both lacks sensory qualities and is single (Ramm, 2017).

If we conceive of the true self along these lines, the result would be rather indeterminate. There would be nothing more to it than what is common to all other selves: a simple and unique existence potentially aware of itself as such. Any identification of the self with a particular property, such as being a human, acting morally, or having been born in a certain place, would be fully irrelevant to the true self. But this seems wrong – or at least too indeterminate. Not only would it be at odds with typical conceptions of the true self, it would also conceptualize the true self in the form of a ghost with no bearing on its environment. This leads us to the thick conception of the true self [compare Galen Strawson’s conception of the self, which differentiates between the self as a distinct mental entity and a subject of experience and the self as an agent, personality and diachronic continuity (Strawson, 1997)]. The thick conception of the true self connects it to certain substantial and moral properties such as being able to form memories or making an existential choice. Hence the thick conception where the true self consists of more determinate characteristics than bare existence is in accordance with how the true self is typically conceived in folk psychology. Is there a specific property or set of properties the self can identify with to become a true self or at least a “truer” self? Can one make a choice or live in a way that does not represent the ideal version of that individual? This certainly seems to be the case. But what is the measure according to which an act or a way of life can be judged as being in accordance with one’s true self? Who or what decides what counts as a proper measure? What is it based on? Where does the true self come from? It will later be discussed how the true self is essentially related to both the past and the future. It will also be suggested that a certain conception of the true self can unite both the thin and thick version of it. Before turning to that, however, we turn to some discussions surrounding the true self in philosophy and psychology.

## The Problem of Radical Subjectivity and Observability of the True Self

Here we will consider the two problems connected to the idea of the true self as identified by Nichols et al. above.

### Radical Subjectivity

As we have seen, the problem of radical subjectivity relates to the notion that how someone conceives of their true self is dependent on what values they have. As we have stated earlier, there are more ways of interpreting what the claim that the true self is “radically subjective” means. It can mean that the true self is based on: (1) something completely arbitrary, (2) an ungrounded existential choice or (3) external factors, such as culture and biology. Although Strohminger et al. do not state explicitly which interpretation they have in mind, we think, based on the examples they give (sexual preference and psychopathy), that the third option is more likely. A person’s sexual preference is rarely considered to be a choice but is rather understood to be based on biology and culture; psychopathy is hardly conceivable as a choice, but, again, is widely believed to be contingent upon biological, cultural or other environmental factors.

This, however, may seem surprising: Does not “radical subjectivity” mean something that involves arbitrariness or some form of creative or spontaneous choice? Since Strohminger et al. speak of the “radical subjectivity” of the true self as tied to what someone prizes or values, there might be some merit to the interpretation of it as being indeterminate in some way (not based on factors external to the self). But then again, the examples they mention point in another direction. So is the critique of the “true self” as radically subjective based on (1) the idea that it is radically arbitrary, random or contingent (what someone *happens* to value) or (2) the idea that the external factors that a person has happened to be exposed to due to the geographical location of their life and their inheritance has determined what they value?

It is highly unlikely that someone would hold the view that what someone values is completely arbitrary, based on something akin to the random result of throwing dice. For example, we value food because of biological needs, friendship because of social needs, and certain ideas because we find them enlightening. However, when we are presented with a moral choice or dilemma or when we are challenged with coming up with a plan for our next steps in life, our choice might seem subjective in the sense that it is creative or ultimately relies on a decision. But if it is creative, this does not mean that it is arbitrary – as we argued above in relation to Foucault. And if it is ultimately based on a decision, this does not mean that we do not have good reasons for acting the way we do, although we might have reasons to act in other ways as well. So the choice itself might be spontaneous, but that does not mean that it is arbitrary in the sense of not being grounded in reasons. And insofar as it is not clear to us what reasons are the best when considering a moral dilemma or committing to a life path, we could regard the choice as creative – but again, such creativity does not have to be arbitrary. What we are left with is the notion that someone’s idea of their true self is radically subjective because it is based on what they happen to value, which in turn is based on the features of their personality. We will consider this in more depth.

Depending on one’s sexual preference or whether one has a personality disorder such as psychopathy, one may conceive of the core of one’s personality differently. This boils down to a claim there are a variety of different conceptions of the self and that therefore how someone defines their true self is subjective. Such a view, however, fails to consider the possibility that one may be right or wrong about their true self. If there were a true self, it would indeed be possible to make such mistakes. We cannot take it for granted that there is no true self based simply on the fact that people value things differently and conceive of their true nature accordingly. Even if I value money and claim that I am affluent, I would be mistaken about this claim if I have no money. Even though people value things differently, and the specific values someone has influence how they conceive of their essential nature, it does not follow that one’s true self is merely an extension of what one happens to value.

However, it is still a significant point that one’s conception of oneself tends to co-vary with one’s cultural background. Could it not be the case that someone’s true self harmonizes with what a specific culture dictates, while someone else in that culture could have a completely different true self; one that runs counter to the common views and values? How would someone know if they were mistaken, i.e. simply influenced by their culture, when it comes to viewing what their true identity is? The true self may indeed be fully individual. How does one uncover it? Perhaps, this is possible exactly by making mistakes or taking on or trying out identities that are not in accordance with one’s true nature.

It seems strange or even wrong to say that by changing one’s identity or taking on a different role, one suddenly lives according to one’s true self. This indeed identifies the true self with the me-self – the true self would be a specific role, identity, job, etc. – which seems counterintuitive; should the true self not be a deep self, the self-as-process? If I change my identity and consider the new identity my true self, it implies that the former identity was a false self. But was it not the case that one aspect of the true self is exactly an underlying identity, one that cannot change simply by changing from one’s surface identity to another? Without such an underlying identity it would not make sense to say that the former identity was a false self, because there is nothing to connect the two identities.

Indeed, the true self may be conceived of as that which unifies different conceptions of the more concrete selves (the me-selves) through a narrative (Polkinghorne, 1991; Gallagher, 2000; Schechtman, 2011), where the variations and mistakes are not necessarily plain errors, but rather essential parts of the process. By manifesting a unity within the different conceptions of the me-self, the true self is also manifested. This manifestation is not necessarily tied to a specific identify, a me-self, being right or wrong, true or false. The measure of the degree of manifestation is the degree of unity created by the processual self-conception. Since the self is also influenced and potentially challenged by different cultures, ethical norms, and worldviews, the unity increases to the extent the different cultures are encompassed, i.e. to the extent that difference is recognized and integrated in the true self.

This capacity of unity may manifest in different ways for different aspects of the true self. Take for example the ethical self, which as pointed out previously, is considered by many to be the true self. Even if one considers the true self to be the ethical self, it does not follow that the true self is radically subjective. What I value may be dependent on a whole range of factors, but that does not mean that the values cannot be judged objectively. There is a long tradition of discussion surrounding the question of whether ethics is objective. However, since there is no consensus on this issue, one cannot say with confidence that values are subjective. Does this mean that the true self is identical to a specific moral set of beliefs? Here, it is helpful to differentiate between different potential layers of the true (ethical) self: (1) the capacity of moral deliberation and action, (2) specific moral views, (3) individual moral or existential choices. At the most fundamental level, a moral self does not consist of a specific set of moral principles and beliefs, but rather of the capacity of ethics, i.e. the capacity of ethical deliberation. Even if one is mistaken about a specific ethical act, the capacity to deliberate offers continuity to the true self. Recognizing that a previous act is wrong is inherently a deepening of the capacity of morality. However, certain acts do not necessarily involve a universal ethical requirement; ethical individualism allows for certain acts being ethical measured only according to the individual (Hegge, 1988). Depending on talent and interest for example might be right for one person to pursue a life as an artist, while wrong for someone else. Furthermore, there may be both general and individual patterns of ethical development that needs to be taken into account. The unity of such patterns, the connection between good and bad actions, failure and success – like the inner coherence of a drama – would be what the true self is.

### Evidence-Insensitivity

Let us look at the argument against observability again:

The true self is, shall we say, evidence-insensitive. Resplendent as the true self is, it is also a bashful thing. Yet people have little trouble imbuing it with a host of hidden properties. Indeed, claims made on its behalf may completely contradict all available data, as when the hopelessly miserable and knavish are nonetheless deemed good ‘deep down’. The true self is posited rather than observed. It is a hopeful phantasm. […] The notion that there are especially authentic parts of the self, and that these parts can remain cloaked from view indefinitely, borders on the superstitious (Strohminger et al., 2017).

There are two related but not identical claims that seem to be inherent in this argument: One is that the true self is in principle unobservable and hence it is an unscientific (superstitious) concept. The other is that what the true self cannot be revised based on evidence, removing it from the domain of science. Both claims will be addressed in the following.

The fact that some properties may be hidden does not in and of itself make the object connected to those properties in principle unavailable to science. Indeed, scientific activity consists of making what is hidden visible, for instance through inventions such as the microscope. However, basing the argument on a contrast hidden/visible implicitly limits the range of inquiry to what we can and cannot *see*, which is unwarranted. Some phenomena, specifically those that unfold in time, are indeed *constitutively dependent on some related properties being unavailable* (“hidden”) *as the phenomenon manifests*. When a phenomenon manifests, something in the previous stage must be removed for a new stage to replace it. In other words, for something to manifest, something that once was, now has to be “hidden.” For someone to say “the true self is not observable,” for example, requires the word “the” to not appear (sound) when “true” is said. In fact, all other words must be “hidden” as well. What is consistent throughout the sentence is the invoked meaning. The meaning is partially invoked by each word and only fully invoked by the whole sentence (which cannot be present as a single instance in time, though perhaps as the retained meaning, something that includes the words and their sequence in a kind of concrete universal, i.e., a concept that is a whole containing its parts in it). Studying time-phenomena such as the self hence requires different methods than those that try to find and measure it at a specific moment in time. The latter approach may find it but only parts of it. Only a narrative that takes the whole into account can be an adequate method for studying the diachronic aspects of the self.

The claim that someone is “good deep down” despite all the evidence to the contrary is harder to counter. A “good” friend who never supports their friends is not a true friend. But is there any point at which someone loses the *capacity* for acting morally or being a good friend? Losing this capacity would also imply a loss of agency and the eligibility to be blamed. The self would be gone or at least not manifest in a basic sense. How could someone therefore provide evidence that the capacity really is absent? If someone always acted in a morally blameworthy way, what we could say, scientifically, i.e. based on observation, is that this person’s true self is evil. However, a single good action would disprove that we have identified an essence. And a case of a person who consistently acts in a morally reprehensible way is hard to conceive. Is it someone who always acts so as to inflict the most pain possible? Is it someone not capable of any form of co-operation? Such a person would seem more like a machine than a human being. Even if we could conceive of such a person, we see no reason to reject the metaphysical possibility that such a person may change their ways. Maybe it would be possible to argue for the existence of evil true selves. Such an argument could very well be interesting but we suggest that for most persons it is possible to discover at least small acts of kindness, which would go to show the presence of a capacity for good. People who have indeed acted in reprehensible or in problematic ways and have changed provide a special area of study in relation to the true self. We take it for granted that such individuals exist. People who go through fundamental change toward good show that simple forms of observation and measurements at specific points in time are not adequate for studying the true self. An approach rather is required that takes long stretches of time into account. Given that there is a capacity for good or at least basic agency, a view that does not take this into account would be less truthful, i.e., less scientific, than a view that does take this into account.

It is still problematic that just as one can always correctly posit the capacity for good, one can also posit the capacity for evil. What is actually representative of one’s true self then would seem to depend on what tendency manifests the most. For this reason, it seems appropriate to have a more abstract conception of the true self, i.e., as something that provides unity to life, and considers the relationship between good and bad acts. Actual human beings will probably never be so bad as to exclusively manifest evil actions and probably never be so good as to never do anything blameworthy. Considering what is good and bad, in the long run, requires historical perspectives. The scientific view is therefore also a view that is continually evolving with time.

One further objection to Strohminger et al. is that reliable methods already exist that objectively measure issues relating to the true self. For example, the ease with which people describe their true self is correlated with life meaning (Schlegel et al., 2009). However, such studies only assess the belief people have about the true self – not its existence. Strohminger et al.’s point is that the belief in the true self is evidence-insensitive in the sense that people are in principle unwilling to revise their view about what they believe their own or someone else’s true self to be. The belief can only be confirmed, not rejected; hence, the true self is a non-scientific concept.

Another response to Strohimger et al.’s skepticism would follow a similar line of argument as Zahavi’s response to Metzinger’s claim that the self is an illusion or a model created by the brain. Zahavi’s response is that the sense of self can be understood to constitute the self, or, in other words, the existence of the self is nothing above and beyond the phenomenal experience of the self (Zahavi, 2005a). We would argue similarly in relation to the true self when faced with reductive arguments. The sense that people have of the existence of a true self can indeed be taken as constitutive of the true self. However, we wish to extend the concept of the true self to include specific life moments or developmental trajectories that manifest the true self, i.e., situations or ways of acting where the true self is not just a sense, but rather something that comes into existence. One could formulate this as an actualization of the potential true self. As we will outline in the next section, the sense of the true self extends not only into the past but also into the future. The existence of the true self in this way transcends time, although it can also appear or manifest for instance during significant life events – such as during Socrates’ trial – where one’s moral character is put to the test.

## Outline of a Comprehensive First-Person Method for Studying the True Self

Understanding the true self as an activity in evolution and a process in metamorphosis involves conceptualizing it in a format that is most likely difficult to be nailed down with conventional, outwardly observable research methods. It is *per se* a first-person phenomenon and hence also requires a first-person mode of enquiry, although it also potentially involves behavioral aspects. It may manifest in a specific behavioral and even biological instantiation. This is however only the outer signature or correlate of the qualia of the phenomenon. This signature can be studied with conventional (e.g., behavioral or even physiological) research methods; the true self *in actu* as a first-person phenomenon, however, cannot be studied in this way. It can only indirectly be inferred from this signature mode of appearance. The approach outlined below can be seen as an extension of first-person approaches to the self that focus on its minimal, synchronous experience as presented by Ramm (2017), who involves for example directing attention to the point from which one looks at the world and investigating this point phenomenologically. The investigation reveals that this point has no visual features, but rather is transparent, single, etc. Through further experiments, one is lead to an experience of a minimal synchronic subject. The focus here is on diachronic aspects of the self, which are essential to study in order to develop a thick account of the self.

One can suspect that the true self can be grasped more by what it *can* become (causa finalis) rather than by what it *has* become (causa efficiens). This has further implications for the way it is studied. As an analogy, take the example of climate change. A small minority of people (mostly climatologists) made the earliest indications while researching subtle and even ambiguous symptoms of complex weather phenomena. For them to persist in their claim and stick to their account, they needed a good sense of trust in their reading and interpretation of the data and early indications. Moreover, they needed a vision of a future that might unfold if things continue in the manner they have developed so far. This was highly unusual and anachronistic at a time where climate change was still outside the conventional thinking style.

In a sense, the challenge that we see in this admittedly far-fetched example is somewhat related to the case of researching the true self. We need to investigate subtle and elusive symptoms to begin with and envision how this true self might unfold if given a chance to manifest and materialize within the constellation of potentials and situational factors with which the individual is endowed. Firmness is needed in envisioning the potentiality of this true self and a sense of trust that it can metamorphose from potential to reality. The moment it manifests as a reality in which to be studied in one way or another will have already crystallized into a given form to be considered a product rather than a process *in actu*. This would be an indication of a sub-component of the true self, not the true self proper.

The method we outline below is an extension of first-person approaches we have developed elsewhere, consisting of small groups of researchers investigating their experience through a series of meetings, note taking, comparison of results and repeated refinement of the experimental tasks that are carried out by the researchers themselves (Weger and Wagemann, 2015; Hackert et al., 2019). For more depth and precision of experiences involved in the descriptions of the events and tasks described below, micro-phenomenological interviews (Petitmengin, 2006) or self-inquiry can be employed.

We propose that a first-person method for studying the true self would include five steps:

1. The first step is developing a conceptual understanding of different possible accounts of what the true self might be. This involves envisioning possible worlds and future realities. Can a sense of one’s true self be evoked through considering scenarios closer or further away from one’s current life and identity? The sphere of the true self is not necessarily only that which is already instantiated but that which is still to come into existence. Without such conceptual guides, we are likely to miss the more subtle traces of the true self as mere background noise.
2. The second step is to consider significant life events (e.g., decisions, moral choices, challenging situations, illnesses, accidents, etc.) where one has the sense of either living up to or failing to live up to one’s true self. Are there common markers of managing and failing to act in accordance with one’s true self? What does the exploration of the sense of living in accordance with the true self reveal about the possible nature of one’s own true self?
3. The third step is to consider the experience of the true self in the present moment. Which of my current properties and identities (gender, job, hair color, nationality, interests, philosophical outlook, etc.) relate to my true self? By employing a version of eidetic variation (Giorgi, 2009), one can change any or all of these identities to see what can possibly be changed before the sense of who one is changes fundamentally. Furthermore, experiments such as described by Ramm (2017) can be employed to access the basic aspects of the synchronic subject. Is the sense of this subject similar to the sense of the true self one has developed to the present day? Additionally, different meditative techniques can be employed in order to heighten the awareness of the minimal self, for example by directing attention away from the awareness of specific thoughts, feelings, bodily sensations, the sense of self, and channeling them toward an awareness of awareness itself. To what extent is the true self connected to the minimal self and pure awareness and to what is it connected to specific properties of the actual/personal self? Can the true self be understood as integrating the minimal and personal self?
4. The fourth step is observing instances during the course of several weeks where one feels more vs. less at one with oneself. How do the instances where one feels more at one with oneself differ from those in which one feels less like oneself? How do such moments relate to the significant life events connected to the true self that were explored in step two?
5. The fifth step is “trusting” the true self into becoming – or one could also say: acting it out. This acting out has both a productive and a receptive side. The unfolding of the activity and getting to know it from inside inherently involve participating in its activity as well as cultivating a sense of receptivity for the inner echo that this activity produces. This fifth step is perhaps the most unusual form of scientific enquiry. This is a reminder that any form of research ultimately strives toward insight and the capacity for action (e.g., in the form of reproducing an effect that nature has created in a scientific physicochemical experiment).

Each step also involves checking one’s understanding of the true self that was developed in the first step. Do any of the further steps lead to a deepening or change of one’s initial conception of the true self? As such, this method involves both philosophical aspects as well as first-person experiments and first-person data gathered from memory. One could refer to such a method as “comprehensive” in that it involves investigating large developmental trajectories, present moment experiences, as well as how they relate to each other. It draws on different first-person methodologies that seem to be adequate for investigating the true self in the way we have presented in the previous sections. It may be noted that the method itself not necessarily presupposes any specific conception of the true self. It is therefore part of the method to reflect continually on what the true self means conceptually. Though the nature of the true self that we have suggested served as a guideline for developing the steps of the method outlined above, it may be that the actual first-person investigations of the true self following this method outlined here will lead to refinements both of the method as well as the account of the true self that we have argued for.

## Conclusion

The basic function of the self is unity. It connects events in time and space into a single continuum of experience. To the extent that this unity is manifested, the true self is manifested. This can happen on different levels: (1) the core self – extending the continuity of the subjective sense of being – linking together orientation in space, time, and situation, and (2) the narrative self – creating unity throughout live events. Though we can say that there cannot be a narrative self without a core self, the converse is also true: The core self cannot actually exist – be aware of itself as a unity – without different moments in time being united within a time-structure. Hence, Zahavi is wrong, in part, in stating that:

[…]…it takes a self to experience one’s life as a story. In order to begin a self-narrative, the narrator must be able to differentiate between self and non-self, must be able to self-attribute actions and experience agency, and must be able to refer to him- or herself by means of the first-person pronoun. All of this presupposes that the narrator is in possession of a first-person perspective (Zahavi, 2005b, p. 114).

Though this is half right, one can also say the opposite: There is no self without a minimal story, a beginning, middle, and end unfolding in time and united across time. However, it is also true that there needs to be an underlying self (unity) to the story. If no time has passed, it cannot be decided whether the self is indeed a self and hence the story/narrative and the minimal, phenomenological self are co-constitutive. In other words, the narrative and core self are co-constitutive and therefore inseparable. Although the latter may become ever more specified and deepened, this cannot happen without the core self. However, as the narrative self becomes more concrete in its various differentiations, the core self expands while not losing any of its being: It is that which is capable of being manifested as all the different concrete identities while not being fully identified with any single one of them. This self, a true self, can potentially be investigated following the methodical approach outlined above.

## Author Contributions

TS has written most of the manuscript. FE has taken part in conceptual development of the manuscript and commented on it. UW has taken part in the conceptual development of the manuscript, commented on it, and written parts of it.

### Conflict of Interest

The authors declare that the research was conducted in the absence of any commercial or financial relationships that could be construed as a potential conflict of interest.
